# Quantifying Morphology of a Differentiating Neuroblastoma Cell Line

**DOI:** 10.17912/micropub.biology.001099

**Published:** 2024-03-19

**Authors:** Jillian Fang, Whitney Kuwamoto, Geanna Miranda, Vanishree Rajagopalan, Tamira Elul

**Affiliations:** 1 Foundational Biomedical Sciences Department, College of Osteopathic Medicine, Touro University California, Vallejo, California, USA; 2 Biological and Pharmaceutical Sciences Department, College of Pharmacy, Touro University California, Vallejo, California, USA

## Abstract

SH-SY5Y neuroblastoma cells are a subclone cell line of SK-N-SH cells derived from neural crest that were originally taken from human bone marrow during a biopsy. Research has shown that these cells can be cultured in vitro to differentiate into mature, neuronal phenotypes such as dopaminergic neurons. Here, we added to these discoveries by establishing a quantitative profile for the SH-SY5Y cells of morphometric features including neurite length, branchpoint numbers, and soma area over the span of 18 days. Overall, we showed that in SH-SY5Y cells neurite length initially decreased followed by a dramatic increase of both neurite length and branching. In contrast, soma area for the SH-SY5Y cells initially increased and then stabilized; followed by a small decrease in size. By determining these morphological changes along various timepoints of SH-SY5Y cell development during the programmed cell differentiation process, we provide a set of baseline data for future mechanistic studies in human-derived neuronal cultures.

**Figure 1. Development of SH-SY5Y cells over an 18-day time period.**
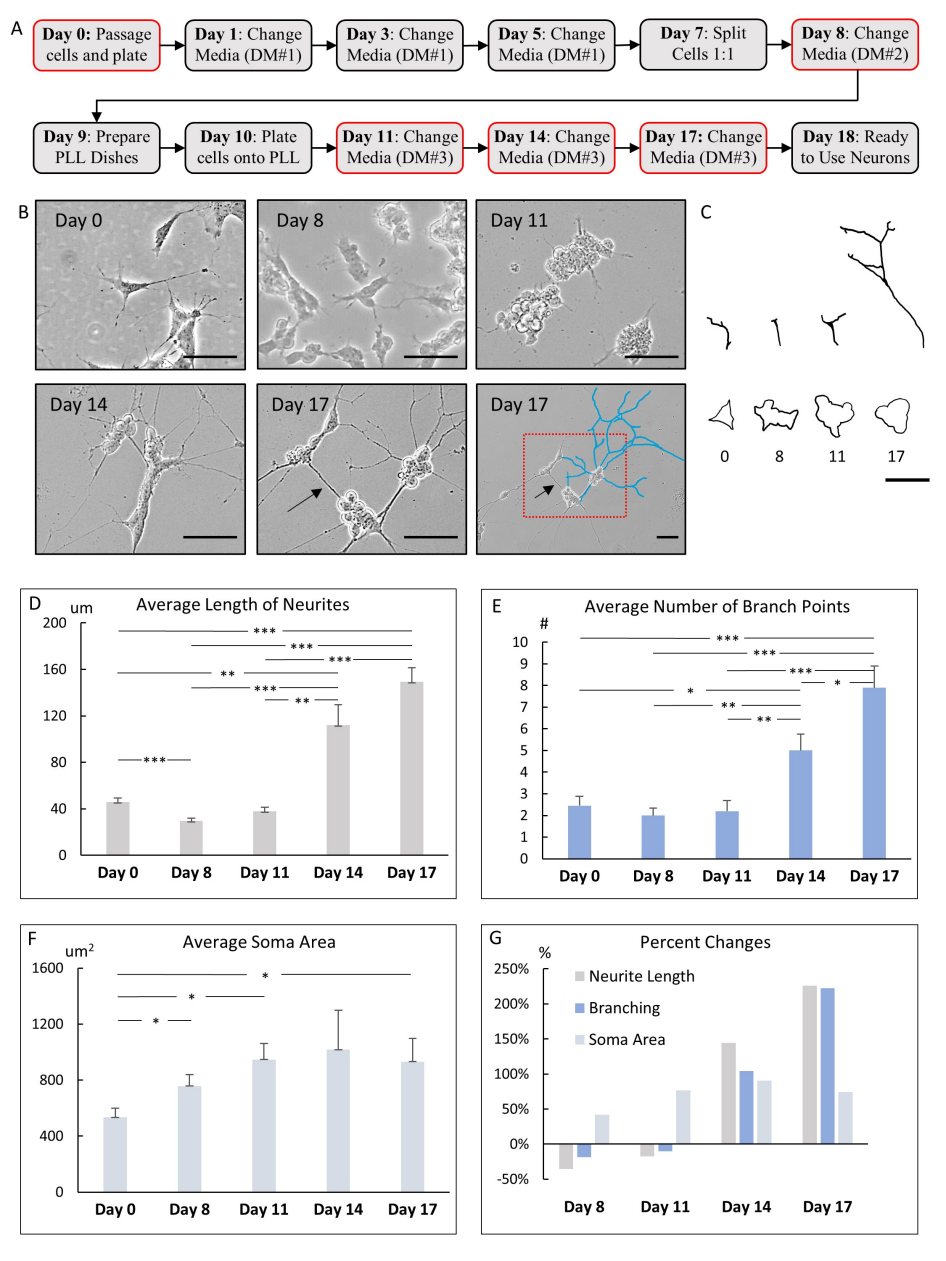
**A)** Timeline for neuronal differentiation protocol where the essential steps are indicated each day, from SH-SY5Y plating to mature cell differentiation. The boxes outlined in red indicate the days the cultures were imaged. **(B** ) Photomicrographs of representative differentiating SH-SY5Y cells after application of different media. Day 0 after application of Basic Growth Media. Day 8 after application of Differentiation Media #1. Day 11 after application of Differentiation Media #2. Day 17 after application of Differentiation Media #3 and becoming a fully mature neuron. Day 17 right panel is a lower magnification view of Day 17 left panel (red box). Day 17 right panel shows some neurites traced in blue. In addition, Day 17 left and right panels show some neurites synapsing together, appearing as one long projection that connects two neurons together (arrows). **(C)** Tracings of representative neurites show overall the change in lengths and complexity from day 0 to day 17. The neurites were shorter on day 8 in comparison to day 0. By day 17, the neurites elongated, and branches emerged. Day 17 shows branching can become complex as the neurites lengthened and began to synapse. Additionally, tracings of a representative soma after growth in basic growth media and specific differentiation medias are shown. There was a gradual increase in the soma area, followed by a small decrease in area in the last days during the differentiation process. Day 0 somas had a polygonal-shaped morphology while day 17 was more rounded. **(D)** Average lengths of neurites over the course of the 18 days of neuronal development. During the initial phase of the differentiation process – day 0 to day 8 – there was a significant decrease in neurite length. At the end of the 18-day observation period, there was a 2.25-fold increase in neurite length because of the introduction of Differentiation Media #3 on day 11. **(E)** Average number of branch points over the course of 18 days **.** The first eight days demonstrated no change or a small decrease in total branch points as neurites became shorter. As neurites increased in length on days 14 and 17, there was a corresponding significant increase in branch points. **(F)** Average soma area over the course of 18 days. From day 0 to day 11, the soma area gradually increased. After the addition of Differentiation media # 3, the soma area stabilized and began to decrease. **(G)** Comparison of percent changes in neurite lengths, branching, and soma area to basic morphology from day 0. During the first eleven days, the average neurite lengths were shorter than those in day 0. The neurites became longer on days 14 and 17. There was a decrease in branch points, contributing to the negative percent change within the first eleven days. As the neurites lengthened, the branching patterns increased by approximately one-fold on day 14 and two-fold by day 17. The overall area of the soma increased until day 11 and then stabilized or began to decrease in size by day 17. (B and C) Scale bars – 50 µm. (D, E, F) *statistical significance with < 0.05. **statistical significance with < 0.01. ***statistical significance with < 0.001.

## Description

SH-SY5Y neuroblastoma cells are derived from human malignant tumor cells and can be differentiated into mature neurons. Both differentiated and undifferentiated SH-SY5Y cells were used in prior studies to investigate mechanisms of neurological disorders such as Parkinson’s disease (PD), Alzheimer’s disease (AD), and pathogenesis of neurotoxicity. Some studies found that Retinoic Acid (RA), phorbol esters, and brain derived neurotrophic factor (BDNF) direct the differentiation of SH-SY5Y cells towards a dopaminergic neuron, rather than an adrenergic or cholinergic neuron. Dopaminergic neurons were used for PD research, whereas undifferentiated SH-SY5Y cells were used to discover new therapies for AD, neuroprotection in PD, and studying neurotoxicity (Xie et al., 2010; Cheung et al., 2009) . The current study aimed to quantitatively assess the neurite development and soma growth of SH-SY5Y-derived neurons. A methods paper, *Differentiation of the SH-SY5Y Human Neuroblastoma Cell Line* system, by Shipley and colleagues (2016) was used as a guide for inducing neuronal differentiation. This process occurred over a period of 18 days. The cells developed in a sequence of four different medias: Basic Growth Media (BGM), Differentiation Media #1 (DM#1), Differentiation Media #2 (DM#2), and Differentiation Media #3 (DM#3). Prior to passaging the cells to a new media, a picture of each plate was captured, these images were examined, and specific measurements were made ( Fig. 1 ).

Our qualitative results were consistent with prior studies (Figs. 1B, 1C). Undifferentiated SH-SY5Y cells had a polygonal ‘epithelial-shaped’ body with very few, short processes (Shipley et al., 2016) . These cells rapidly divided, clustered into groups, and expressed immature neuronal markers and truncated processes (Kovalevich and Langford, 2013) . In contrast, differentiated SH-SY5Y cells showed decreased proliferation and extended long neurites (Shipley et al., 2016) . Healthy differentiated SH-SY5Y cells extended numerous neurite branches that connected to other nearby projections with clear distance from each other (Shipley et al., 2016) . A prior paper quantitatively analyzed the neurite extension and branching of differentiated SH-SY5Y neuroblastoma cells on day 10 of the differentiation protocol (Thomson et al., 2022) . However, the period of observation in this study was limited to 24 hours and the media concentrations differed from our procedure (contained 10% hiFBS for all 10 days and lacked BNDF). Our new findings included quantitative results related to SH-SY5Y cell neurites, branches, and soma over 18 days of differentiation with four different media (Shipley et al., 2016) . Initially our findings show that the differences in neurotrophic factors produced shorter neurites, followed by an elongation and branching period (Figs. 1D, 1E, 1G). In contrast, the soma area initially increased, and subsequently remained the same (or decreased in) size (Figs. 1F, 1G).

**DM#1:** From day 0 to day 8, the recurrent application of DM#1 significantly decreased average neurite length by 35% (0 – 8 days; 47.9 ± 3.43 mm in BGM (n = 20 neurites), 29.7 ± 2.15 mm in DM#1 (n = 38 neurites), p = 3.40E-4, Figs.1D, 1G). This indicated the neuroblastoma neurites were shortening during initial phases of serum-starvation. In addition, at day 0, the neurite projections had few branches, and by Day 8, after time in the DM#1, there was an insignificant 18% decrease in the number of branches (0 – 8 days; 2.5 ± 0.44 branchpoints for BGM (n = 20 neurites), 2.0 ± 0.33 branchpoints for DM#1 (n = 10 neurites), p = 0.421, Figs. 1E, 1G). In contrast, the soma grew in DM#1 with a 42 % increase in mean area from day 0 to day 8 (0 – 8 days; 535.1 ± 63.30 mm ^2^ for BGM (n = 11 neurons), 758 ± 82.24 mm ^2^ for DM#1 (n = 25 neurons), p = 0.0391, Figs. 1F, 1G).

**DM#2:** The effects of DM#2 differed from DM#1. On day 11, there was a non-statistically significant 17 % decrease in average length of neurites from initial neurite length measurements at day 0 (0 – 11 days; 45.9 ± 3.43 mm in BGM (n = 20 neurites), 37.9 ± 3.50 mm in DM#2 (n = 27 neurites), p = 0.112, Figs. 1D, 1G). Because the decrease in neurite length at day 8 compared to day 0 was significant (see above), this suggests that by day 11, the projections may be beginning to increase in length. The small increase in extension of the neurites on day 11 could be a result of passaging the cells onto the electrostatically active poly-L-lysine (PLL) coated plates on day 10. Prior studies found that electrostimulation promotes nerve growth factor (NGF) induced neurite outgrowth signaling (Yavin, 1977) . Another possible reason for the small increase in neurites from day 8 to day 11 is because the cells underwent regular medium change. The addition of fresh medium has been correlated with an increase in the growth factor BDNF (Thomson et al., 2022) . The small increase in growth of the neurites on day 11 contributed to insignificant changes in branching patterns (Figs. 1E, 1G). There was an insignificant 10% decrease in the number of branchpoints from days 0 to 11 (0 – 11 days; 2.5 ± 0.44 branch points for BGM (n = 20 neurites), 2.2 ± 0.49 branch points DM#2 (n = 10 neurites), p = 0.707; Fig. 1E, 1G). In contrast, the addition of DM#2 (including 1% hiFBS) allowed the soma to continue to increase in mean area by 72% (0 – 11 days; 535.1 ± 63.3 mm ^2^ for BGM (n = 11 neurons), 947.1 ± 114.1 mm ^2^ for DM#2 (n = 36 neurons), p = 0.0028; Figs. 1F, 1G).

**DM#3:** SH-SY5Y neurite length, branching and soma area were all changed significantly with the complete removal of DM#2 (containing hiFBS) and introduction of DM# 3 (containing BDNF and db-cAMP). On day 14, we observed a dramatic increase in neurite lengths by 145% (0 – 14 days; 45.9 ± 3.43 mm for BGM (n = 20 neurites), 112.1 ± 17.47 mm for DM#3 on day 14 (n = 13 neurites), p = 0.0026, Figs. 1D, 1G). These findings are further supported by our data on day 17 where neurite extension shows a 226% increase compared to day 0 (0 – 17 days; 45.9 ± 3.43 mm for BGM (n = 20 neurites), 149.4 ± 11.96 mm for DM#3 on day 17 (n = 38 neurites), p = 1.73E-10, Figs. 1D, 1G). The complete removal of DM#2 also promoted structural plasticity by increasing the number of neurite branches. We observed a 104% increase in branches from day 0 to day 14, and a 222 % increase in branches from day 0 to day 17 (0 – 17 days; 2.5 ± 0.44 branchpoints for BGM (n = 20 neurites), 5 ± 0.335 branchpoints for DM#3 on day 14 (n = 10 neurites), 7.9 ± 1.0 branchpoints for DM#3 on day 17 (n = 10 neurites), p = 0.00029, Fig. 1E, 1G). This is likely due to the addition of BDNF which has previously been shown to promote dendritic and axonal branching, along with synapse development and plasticity, essential features of mature neurons (Palasz et al., 2020; Cohen-Cory et al., 2022) . While in DM#3 the SH-SY5Y cells’ soma area first increased in size by 90% on day 14 compared to day 0 (0 – 14 days; 535.1 ± 63.3 mm ^2^ for BGM (n = 11 neurons), 1018.1 ± 280.6 microns ^2^ for DM#3 on day 14 (n = 13 neurons), p = 0.117, Figs. 1F, 1G). However, on day 17, there was a smaller 75% increase in mean neuron cell body area compared to day 0 (0 – 17 days; 535.1 ± 63.3 mm ^2^ for BGM (n = 11 neurons), 932.0 ± 264.3 microns ^2^ for DM#3 on day 17 (n = 21 neurons), p = 0.0033, Figs. 1F, 1G). This may indicate that the soma area is beginning to stabilize or decrease in size as the SH-SY5Y cells continue to differentiate. The pathways involved in regulating soma size require further investigation as the mechanism is not well understood.

Overall, we observed that as the SH-SY5Y cells matured, they first showed a decrease in neurite length followed by a significant increase in both elongation and branching of neurites. In contrast, during the differentiation process of SH-SY5Y cells, the soma area first increased in size, but then stabilized, and by the end, may have begun to decrease, in size. These results provide fundamental data on the morphological differentiation of SH-SY5Y cells that can serve as baseline parameters for future mechanistic studies.

## Methods

SH-SY5Y cells were grown *in vitro* to produce neurons for further experiments. Four different media containing a variety of factors were used to achieve differentiated neurons. Each media had different concentrations of each factor and the time the cells grew in specific media also varied ( Fig. 1A ). Overall, these conditions included gradual reduction of heat-inactivated fetal bovine serum (hiFBS) to halt cell proliferation (Chelladurai et al., 2021), along with addition of RA and neurotrophic agents such as BDNF to shift the cells to differentiation (Shipley et al., 2016) .

Beginning on day 0, a basic growth media (BGM) with 15% hiFBS was used to initiate the growth of SH-SY5Y cells. On day 1, DM#1 with 2.5% hiFBS was used to replace the BGM; DM#1 was replaced by fresh DM#1 every day until day 8. On day 8, DM#1 was replaced with DM#2 which contains only 1% hiFBS. RA was also consistently used in the DM#1 and DM#2 to inhibit growth and development of the epithelial-phenotype cells (Sidell et al., 1983) . RA, a lipophilic metabolic of vitamin A, is commonly used as a trophic factor to induce neurite growth by binding to transcription factors and inducing gene expression (Teppola et al., 2016) . The mechanism responsible for this includes arresting cell cycle progressions out of G0/G1, increasing cyclin-dependent kinase (CDK) inhibitors p21 and p27, increasing anti-apoptotic proteins Bcl-2 and Bcl-xL, and enhancing PI3K/AKT activity to aid neurite development and differentiation (Shipley et al., 2016) . Trypsin was also included to reduce the confluency of the cells on days 0, 7, and 10. The media was replaced daily with fresh DM#2 until day 11, when DM#3 was added to the cultures. In preparation for the addition of DM#3, on day 10, plates were coated with poly-L-lysine to ensure the remaining few neuroblastoma cells would adhere to the plate. DM#3 did not contain any hiFBS but introduced a new growth factor: BDNF in combination with db-cAMP. BDNF is a neurotrophic that activates the Trk family of receptor tyrosine kinases and p75NTR, a member of TNF receptor superfamily (Huang et al., 2001) . This pathway inhibits cell apoptosis, neurotoxicity, and oxidative damage and ensures cell differentiation, including neurite branching (Palasz et al., 2020) . db-cAMP, a cyclic nucleotide derivative of cAMP, promotes axon regeneration. It is given in combination with BDNF to increase the survival and growth of dopaminergic neurons, as BDNF alone cannot increase the survival rate of these neurons (Lara et al., 2003) .

During the process for differentiation of SH-SY5Y cells (18 days), images were captured prior to each passage ( Fig. 1A ). For instance, day 8 images were consistent with effects of only DM#1. The cultures were examined through a microscope and an image was captured at 20x magnification. Two microscopes were used for imaging: An inverted Nikon Eclipse TS100 with a Nikon 20x/0.40 Ph1 ADL objective and a Lumenera Infinity Lite B CMOS camera attachment was used to capture images of cells at days 0 and 8. This camera was compatible with the Lumenera Infinity Analyze and Capture software that transferred the images to a computer. On days 11, 14, and 17, a Keyence Digital microscope was used to capture the morphology of more mature and differentiated neuroblastoma cells.

Image processing and analysis was done in ImageJ (1.53t / Java 1.8.0_345 64-bit; NIH). We calibrated the ImageJ software with measurements in micrometers prior to analyzing the images captured of the live cells (Mucci et al., 2022) . All images were analyzed for neurite length, branchpoint numbers and soma area. Neurite length was measured using the segmented line tool. 15 – 40 neurites were measured per day, depending on how many cells were visible for each day. ImageJ was not used for the quantification of the branch points. We were able to assess the number of branch points per neurite by directly scoring them from the images. The sample size for branch points was 20 neurites for day 0 and 10 neurites for all other days. Synapsing neurites and overlapping branching patterns were more difficult to distinguish in later days ( Fig. 1B last panel ), thus contributing to the lower sample size, and possibly resulting in an overcount or undercount of branch point numbers. Unlike neurites, the cell body was easier to observe and measure. The cell bodies were outlined and measured using the freehand tool in ImageJ. A sample size of 15 – 25 somas for days 0 and 8 were used, while later days had a sample size of 15 – 40 somas. To test for statistical significance between morphometric measurements on day 0 and subsequent differentiation days, two-tailed t-tests were used ( Fig. 1D, E, F ).

## Reagents

**Media Recipes**

**Table:** 

**Basic Growth Media**		
**Component**	**Volume for 500 ml**	**Dilution**
EMEM	415 ml EMEM	
15% hiFBS	75 ml hiFBS	
1x Pen/Strep	5 ml Pen/Strep	1:100
2 mM Glutamine	5 ml Glutamine	1:100
*Keep for 6 weeks maximum		
**Differentiation Media #1**		
**Component**	**Volume for 50 ml**	**Dilution**
EMEM	48 ml EMEM	
2.5% hiFBS	1.3 ml hiFBS	
1x Pen/Strep	500 μl Pen/Strep	1:100
2 mM Glutamine	500 μl Glutamine	1:100
10 μM RA	100 μl RA (5mM stock)	1:500
*Keep for 2 weeks maximum and add RA immediately prior to use		
*Do not keep extra media once RA is added - RA is unstable		
**Differentiation Media #2**		
**Component**	**Volume for 50 ml**	**Dilution**
EMEM	49 ml EMEM	
1% hiFBS	500 μl hiFBS	
1x Pen/Strep	500 μl Pen/Strep	1:100
2 mM Glutamine	500 μl Glutamine	1:100
10 μM RA	100 μl RA (5mM stock)	1:500
*Keep for 2 weeks maximum and add RA immediately prior to use		
*Do not keep extra media once RA is added - RA is unstable		
**Differentiation Media #3**		
**Component**	**Volume for 50 ml**	**Dilution**
Neurobasal	47 ml Neurobasal	
1x B-27	1 ml B-27 (50X stock)	1:50
20 mM KCl	1 ml KCl (1M stock)	1:50
1x Pen/Strep	500 μl Pen/Strep	1:100
2 mM GlutamaxI	500 μl GlutamaxI (100x stock)	1:100
50 ng/ml BDNF	250 μl BDNF stock (10 μg/ml)	1:200
2 mM dibutyryl cyclic AMP (db-cAMP)	100 μl db-cAMP (1M stock)	1:500
10 μM RA	100 μl RA (5 mM stock)	1:500
*Keep for 2 weeks maximum and add RA immediately prior to use		
*Do not keep extra media once RA is added - RA is unstable		

(Shipley et al., 2016)
